# Case report: Complete pathological admission in N3 unresectable locally advanced lung adenocarcinoma with a novel INTS10-ALK and EML4-ALK fusion after neoadjuvant crizotinib

**DOI:** 10.3389/fonc.2023.1104910

**Published:** 2023-03-29

**Authors:** Xiaoqian Zhai, Ting Wang, Yiyun Lin, Jiabi Zhang, Yuqing Wang, Weiya Wang, Qinghua Zhou, Daxing Zhu

**Affiliations:** ^1^ Lung Cancer Center, West China Hospital, Sichuan University, Chengdu, China; ^2^ Graduate School of Biomedical Sciences, MD Anderson Cancer Center UT Health, Houston, TX, United States; ^3^ Department of Nutrition and Integrative Physiology, College of Health, University of Utah, Salt Lake City, UT, United States; ^4^ Graduate School of Biomedical Sciences, Baylor College of Medicine, Houston, TX, United States; ^5^ Pathology Department, West China Hospital, Sichuan University, Chengdu, China

**Keywords:** stage IIIB-N3, INTS10-ALK and EML4-ALK, neoadjuvant, crizotinib, complete pathologic response, lung cancer, case report

## Abstract

**Background:**

Although anaplastic lymphoma kinase tyrosine kinase inhibitors (ALK-TKIs) have impressive response in advanced lung adenocarcinoma with anaplastic lymphoma kinase (ALK) fusion, no guidelines point to the potential benefits of neoadjuvant ALK-TKIs for N3 unresectable locally advanced lung cancer. Current ongoing clinical trials mainly focus on the efficacy of neoadjuvant ALK-TKIs in resectable locally advanced lung cancer and ignore the role of neoadjuvant ALK-TKIs in N3 unresectable locally advanced lung cancer.

**Materials and methods:**

We report a lung cancer case with a novel INTS10-ALK and EML4-ALK rearrangement that achieved complete pathologic response to neoadjuvant crizotinib. We conducted molecular pathologic analysis by using next-generation sequencing (NGS). Genomic DNA was extracted from formalin-fixed paraffin-embedded (FFPE) samples and profiled using a capture-based targeted sequencing panel consisting of 56 lung cancer-related genes.

**Results:**

Our study reported a patient with stage IIIB-N3 lung adenocarcinoma with an unreported dual ALK rearrangement (INTS10-ALK and EML4-ALK) who received 5 months of crizotinib, followed by R0 right upper lobectomy, achieving complete pathological response (ypT0 ypN0). No recurrence of the tumor was found for 3 years postoperatively.

**Conclusion:**

The case supports the strategy of neoadjuvant ALK inhibitors for N3 unresectable locally advanced lung cancer, expanding the spectrum of treatment of stage IIIB-N3 lung cancer.

## Introduction

Stage III non-small cell lung cancer (NSCLC) is a class of diseases with large heterogeneity in clinical manifestations and treatment methods (1). In particular, for stage IIIB-N3 NSCLC, the National Comprehensive Cancer Network (NCCN) recommends concurrent radical chemoradiotherapy and follow-up with immunotherapy. However, stage IIIB-N3 NSCLC only has median progression-free survival (PFS) of approximately 16.8 months, which is based on the data of the PCIFIC study (2). Improving the PFS and overall survival (OS) of patients with stage IIIB-N3 NSCLC is an urgent issue in clinical research.

Although anaplastic lymphoma kinase tyrosine kinase inhibitors (ALK-TKIs) have impressive response in advanced lung adenocarcinoma with anaplastic lymphoma kinase (ALK) fusion, no guidelines point to the potential value of neoadjuvant targeted therapy in stage III ALK fusion NSCLC. Currently, only one retrospective study aimed at N2 resectable locally advanced lung cancer found that neoadjuvant crizotinib could achieve 90.9% (10/11) partial response and 18.1% (2/11) completely pathological remission (3). In addition, several clinical trials that studied the efficacy of neoadjuvant second-generation ALK-TKIs (such as neoadjuvant alectinib) in resectable locally advanced lung cancer have been ongoing (4). However, the efficacy and role of neoadjuvant ALK-TKIs in N3 unresectable locally advanced lung cancer are still undetermined.

Therefore, our study reported a patient with stage IIIB-N3 lung adenocarcinoma with a novel dual ALK fusion (INTS10-ALK and EML4-ALK) who achieved complete pathological response after receiving neoadjuvant crizotinib. We hope that emerging data of neoadjuvant second-generation TKIs in stage IIIB NSCLC will be reported in the future, expanding the spectrum of treatment of stage IIIB-N3 lung cancer.

## Case presentation

**Figure 1:**
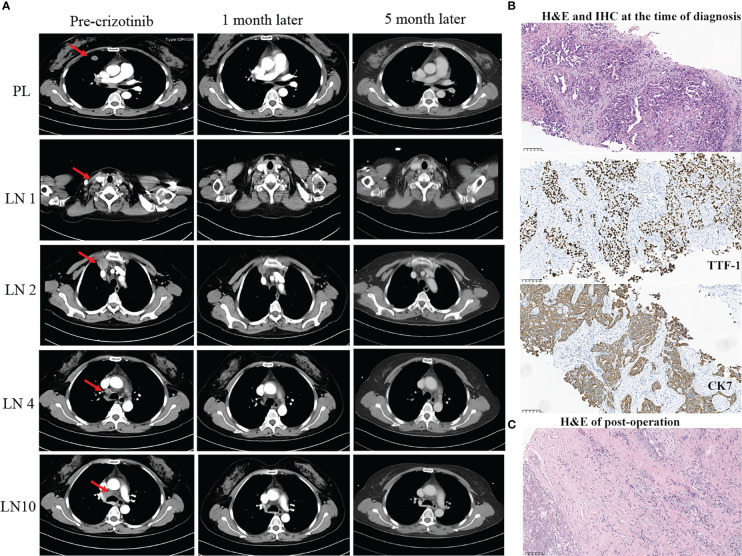
Summary of radiology, pathology, and next-generation sequencing (NGS) results **(A–C)**. **(A)** Radiological evaluation of the patient before and after neoadjuvant crizotinib for 1 and 5 months. PL, primary lung cancer; LN, lymph nodes. **(B)** H&E and IHC staining. The result demonstrated at the time of diagnosis of NSCLC (scale bar, 100 μm). **(C)** H&E staining post-operation. The result showed no residual viable cancer cells after adjuvant crizotinib treatment (scale bar, 100 μm). H&E, hematoxylin and eosin; IHC, immunohistochemistry.

**Figure 2:**
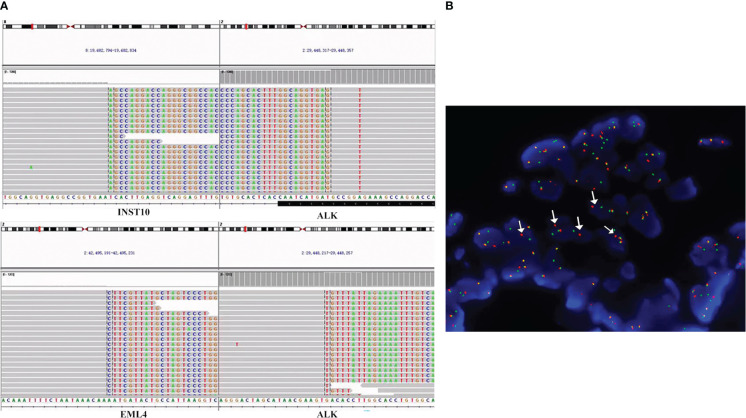
ALK fusions detected by the targeted NGS assay and FFPE FISH. **(A)** Sequencing reads of INTS10-ALK fusion and EML4-ALK fusion rearrangements at the time of diagnosis. Taking the INTS10-ALK fusion as an example, the top part of the picture describes the position of the gene on the chromosome. INTS10 on chromosome 8 (8 exons) fused with ALK on chromosome 2 (20 exons). In the bottom part, each row represents a read. Furthermore, for each row, the normal bases of each gene in each row are in gray, and the colored bases belong to another gene. For example, the gray bases in INS-10 are normal bases, and the colored mutilation bases in INST-10 belong to ALK. Similarly, the gray bases in ALK are normal bases, and the colored mutation bases in ALK are part of INST-10 bases. The same principle can annotate the sequencing results of EML4-ALK. **(B)** FISH result. Using ALK break-apart probe shows ALK rearrangements. Green signals represent the 5′-ALK gene, red signals represent 3′-ALK, and yellow signals represent intact ALK gene. Therefore, arrows point to ALK rearrangements because of the separation of red and green signals. FISH, fluorescence *in situ* hybridization; NGS, next-generation sequencing; FFPE, formalin-fixed paraffin-embedded.

A 54-year-old Chinese woman, without a history of smoking, presented with a palpable right supraclavicular lymph nodule in April 2019 without local redness and tenderness. The patient was worried and came to our hospital because her father has died of lung cancer several years ago. A chest computed tomography (CT) scan was performed and revealed a lobulated nodule in the right upper lobe (22 mm * 15 mm) and multiple enlarged lymph nodes in the right neck and mediastinum (**Figure 1A**). Further, a biopsy of the right supraclavicular lymph node was performed and showed poorly differentiated metastatic lung adenocarcinoma (**Figure 1B**). In addition, molecular pathologic analysis using next-generation sequencing (NGS) was conducted. Genomic DNA was extracted from formalin-fixed paraffin-embedded (FFPE) samples and profiled using a capture-based targeted sequencing panel consisting of 56 lung cancer-related genes (Lung Core, Burning Rock Biotech, Guangzhou, China). Sequence data were mapped to the reference human genome (hg19) using Burrows-Wheeler Aligner version 0.7.10. Structural rearrangement was analyzed using an in-house algorithm markSV (Burning Rock Biotech). The analytical sensitivity of the company’s NGS platform is >95%; analytical specificity is >99%; mean sequence depth is 886×. Therefore, the results of sequencing are reliable. As a result, an unreported dual ALK arrangement—INTS10-ALK fusion (I18: A20, 25.85%) and EML4-ALK fusion (E6: A20, 12.64%)—was detected (**Figure 2A**). Meanwhile, fluorescence *in situ* hybridization (FISH) of ALK was conducted and confirmed ALK rearrangement (**Figure 2B**). As such, the patient was diagnosed with right lung adenocarcinoma with mediastinal and right supraclavicular lymph node metastasis (stage IIIB, cT1cN3M0). Crizotinib was then administrated to the patient in May 2019. Five months later, a re-examination using chest CT detected that the size of a nodule in the right lung had clearly decreased, with dimensions of approximately 8 mm * 5 mm (**Figure 1A**). On 15 October 2019, the patient underwent right upper lobectomy plus systematic lymph node dissection plus right supraclavicular lymph node dissection. Postoperative pathological examination showed the infiltration of inflammatory cells and proliferation of fibrous tissue. There were no definite tumor residues in the primary lesion and lymph nodes (ypT0 ypN0) (**Figure 1C**). After the operation, the patient had poor wound repair, which may be related to the preoperative ALK-TKI administration. Overall, the stage IIIB-N3 patient achieved complete pathological remission after neoadjuvant crizotinib therapy. Postoperatively, the patient continued to take crizotinib (250 mg b.i.d.) for 3 years with no recurrence of the tumor.

## Discussion

Some previous studies found that NSCLC patients with rare ALK rearrangement were not responsive or even resistant to ALK-TKIs (5–7). This study first reports a new dual ALK fusion (INTS10-ALK and EML4-ALK) in N3 unresectable locally advanced lung adenocarcinoma with complete response after crizotinib neoadjuvant treatment. Ou SI et al. summarized and discovered 90 ALK fusion new partners, such as NPM1, BCL11A, and BIRC6 (7). Finding a new partner for ALK rearrangement is still gaining attention. Based on previous literature and summaries, ALK arrangement could be divided into five types: EML4-ALK alone fusion (8), none-EML4-ALK alone fusion, non-reciprocal/reciprocal ALK translocation (6, 9), dual ALK fusion (10), and intergenic ALK fusion (11). Among them, Nongyang et al. found that non-reciprocal/reciprocal ALK translocation and dual ALK fusion in advanced patients had the worst mean PFS (6.1 months) and the highest risk of brain metastasis when compared to the other four types (6). In this study, although INTS10-ALK and EML4-ALK are a dual ALK fusion, the stage IIIB-N3 patient with this arrangement not only has the opportunity for radical surgical treatment but also has attained more than 3 years of PFS due to neoadjuvant crizotinib, which is satisfactory.

Overall, the use of neoadjuvant targeted therapy strategies in N3 unresectable locally advanced lung adenocarcinoma is favorable. In particular, the second-generation TKI alectinib, a highly selective ALK inhibitor with a high response rate and superior efficacy in preventing and treating brain metastasis, is widely used currently in advanced NSCLC (12). There currently exist two clinical case reports reporting the feasibility and safety of alectinib as a neoadjuvant drug (13, 14). Meanwhile, the ALNEO trial is also enrolling patients to study the activity and safety of neoadjuvant alectinib (4). However, the above studies are all focusing on N2 resectable locally advanced NSCLC. At present, our study is the first one to report a patient with N3 unresectable locally advanced NSCLC who achieved pathological remission after neoadjuvant crizotinib treatment with unreported ALK fusion. Emerging evidence is needed in the targeted neoadjuvant area of unresectable N3 NSCLC in the future.

## Data availability statement

The original contributions presented in the study are included in the article/supplementary material. Further inquiries can be directed to the corresponding authors.

## Ethics statement

The studies involving human participants were reviewed and approved by West China Hospital, Sichuan University ethics committee. The patients/participants provided their written informed consent to participate in this study. Written informed consent was obtained from the individual for the publication of any potentially identifiable images or data included in this article.

## Author contributions

XZ and TW: conceptualization, methodology, and writing—original draft. YL: writing—reviewing and editing. JZ and YW: data curation and figure preparation. WW, DZ, and QZ: writing—reviewing and editing, and resource. All authors contributed to the article and approved the submitted version.

## References

[1] Daly ME Singh N Ismaila N Antonoff MB Arenberg DA Bradley J. Management of stage III non-Small-Cell lung cancer: ASCO guideline. J Clin Oncol (2022) 40(12):1356–84. 10.1200/JCO.21.02528 34936470

[2] Antonia SJ Villegas A Daniel D Vicente D Murakami S Hui R. Durvalumab after chemoradiotherapy in stage III non–Small-Cell lung cancer. New Engl J Med (2017) 377(20):1919–29. doi: 10.1056/NEJMoa1709937 28885881

[3] Zhang C Li SL Nie Q Dong S Shao Y Yang XN. Neoadjuvant crizotinib in resectable locally advanced non-small cell lung cancer with ALK rearrangement. J Thorac Oncol (2019) 14(4):726–31. doi: 10.1016/j.jtho.2018.10.161 30408570

[4] Leonetti A Minari R Boni L Gnetti L Verzè M Ventura L. Open-label, single-arm, multicenter study to assess the activity and safety of alectinib as neoadjuvant treatment in surgically resectable stage III ALK-positive NSCLC: ALNEO trial. Clin Lung Cancer (2021) 22(5):473–7. doi: 10.1016/j.cllc.2021.02.014 33762169

[5] Tian P Liu Y Zeng H Tang Y Lizaso A Ye J. Unique molecular features and clinical outcomes in young patients with non-small cell lung cancer harboring ALK fusion genes. J Cancer Res Clin Oncol (2020) 146(4):935–44. doi: 10.1007/s00432-019-03116-6 PMC11804450 31894386

[6] Zhang Y Zeng L Zhou C Li Y Wu L Xia C. Detection of Nonreciprocal/Reciprocal ALK translocation as poor predictive marker in patients with first-line crizotinib-treated ALK-rearranged NSCLC. J Thorac Oncol (2020) 15(6):1027–36. doi: 10.1016/j.jtho.2020.02.007 32112982

[7] Ou SI Zhu VW Nagasaka M. Catalog of 5' fusion partners in ALK-positive NSCLC circa 2020. JTO Clin Res Rep (2020) 1(1):100015. doi: 10.1016/j.jtocrr.2020.100015 34589917 PMC8474466

[8] Soda M Choi YL Enomoto M Takada S Yamashita Y Ishikawa S. Identification of the transforming EML4-ALK fusion gene in non-small-cell lung cancer. Nature (2007) 448(7153):561–6. doi: 10.1038/nature05945 17625570

[9] Zhai X Wu Q Pu D Yin L Wang W Zhu D. Case report: A novel non-reciprocal ALK fusion: ALK-GCA and EML4-ALK were identified in lung adenocarcinoma, which may respond to alectinib adjuvant-targeted therapy. Front Oncol (2021) 11:782682. doi: 10.3389/fonc.2021.782682 35070986 PMC8767047

[10] Zhang Y Zeng L Yang N Jiang T Zhou C. P2.14-51 dual ALK fusion partners as poor predictive marker in first line crizotinib treated ALK rearranged non-small cell lung cancer. J Thorac Oncol (2019) 14(10, Supplement):S849–S50.

[11] Zhai X Wu Q Zeng Z Suo J Lin F Zhou Q. OFCC1-ALK (Ointergenic: A20): A novel OFCC1 intergenic region-ALK fusion identified from a lung adenocarcinoma patient. Lung Cancer (2021) 153:171–3. doi: 10.1016/j.lungcan.2020.12.034 33483163

[12] Peters S Camidge DR Shaw AT Gadgeel S Ahn JS Kim D-W. Alectinib versus crizotinib in untreated ALK-positive non–Small-Cell lung cancer. New Engl J Med (2017) 377(9):829–38. doi: 10.1056/NEJMoa1704795 28586279

[13] Zhang C Yan L-X Jiang B-Y Wu Y-L Zhong W-Z. Feasibility and safety of neoadjuvant alectinib in a patient with ALK-positive locally advanced NSCLC. J Thorac Oncol (2020) 15(6):e95–e9. doi: 10.1016/j.jtho.2019.12.133 32471573

[14] Hu Y Ren S Wang R Han W Xiao P Wang L. Case report: Pathological complete response to neoadjuvant alectinib in a patient with resectable ALK-positive non-small cell lung cancer. Front Pharmacol (2022) 13:816683. doi: 10.3389/fphar.2022.816683 35873553 PMC9299059

